# G9a Suppression Alleviates Corneal Neovascularization through Blocking Nox4-Mediated Oxidative Stress

**DOI:** 10.1155/2020/6983268

**Published:** 2020-03-12

**Authors:** Shanshan Wan, Wanju Yang, Yumiao Pan, Zhuoqun Rao, Yanning Yang

**Affiliations:** Department of Ophthalmology, Renmin Hospital of Wuhan University, Wuhan 430060, China

## Abstract

**Background:**

G9a, a well-known methyltransferase, plays a vital role in biological processes. However, its role in corneal neovascularization (CoNV) remains unclear. *Methods. In vitro* and *in vivo* models were assessed in hypoxia-stimulated angiogenesis and in a mouse model of alkali burn-induced CoNV. Human umbilical vein endothelial cells (HUVECs) were cultured under hypoxic conditions and different reoxygenation times to identify the molecular mechanisms involved in this process.

**Results:**

In this study, we found that G9a was positively related to corneal alkali burn-induced injury. Inhibition of G9a with BIX 01294 (BIX) alleviated corneal injury, including oxidative stress and neovascularization *in vivo* models were assessed in hypoxia-stimulated angiogenesis and in a mouse model of alkali burn-induced CoNV. Human umbilical vein endothelial cells (HUVECs) were cultured under hypoxic conditions and different reoxygenation times to identify the molecular mechanisms involved in this process.

## 1. Introduction

Angiogenesis is an essential biological process that plays a vital role in the tumorigenesis, diabetic retinopathy, and macular degeneration. In the eye, the cornea is a transparent tissue in the front of the eye and plays important roles in vision and light refraction. Moreover, the cornea acts as a mechanical barrier to provide protection against external injuries, including toxicants and microorganisms. Typically, the cornea is transparent and contains antiangiogenic factors that sustain the avascular status [1]. However, pathological changes in the cornea will occur after exposure to immunological disease, chemical burns, trauma, and infection [2], leading to an elevation of proangiogenic gene expression [3]. Corneal neovascularization (CoNV) is characterized by growth of neonatal blood vessels from the limbus of the cornea toward the clear centre, which results in corneal opacity. Meanwhile, corneal oedema induced by CoNV reduces the transparency of the cornea and further influences visual acuity [4]. In the United States of America, approximately 1.4 million individuals suffer from vision impairment secondary to abnormal blood vessel growth [5], presenting a great challenge to ophthalmologists.

Epigenetic regulation, including histone modifications, DNA methylation, and microRNA expression, play vital roles in biological processes such as cell proliferation, apoptosis, inflammation, and neovascularization by regulating transcriptional activity [6–9]. Methylation of lysine 9 of histone H3 (H3K9) is related to euchromatin gene silencing through heterochromatin protein-1 binding [10]. G9a is the second histone methyltransferase found in mammals, and it plays a vital role in triggering the trimethylation of histone H3 at lysine 9 (H3K9me1 and H3K9me2), which is involved in the progression of a variety of biological processes [11]. A previous study demonstrated that G9a histone methyltransferase activity in retinal progenitors is essential for proper differentiation and survival of mouse retinal cells [12]. Also, another study reported that inhibition of G9a by BIX 01294 led to the suppression of cell proliferation, migration, and invasion in in vitro experiments [13]. However, the role and underlying mechanism of G9a in CoNV has not been elucidated.

It is well known that oxidative stress plays an important role in chemical burn-induced corneal damage, and oxidative stress is characterized by increased reactive oxygen species (ROS) production [14]. ROS consists of highly reactive molecules, including hydroxyl radical, hydrogen peroxide (H_2_O_2_), and superoxide radical. ROS regulates in cellular homeostasis under physiological conditions, whereas pathologically high levels of ROS are related to cell death, inflammation, and apoptosis. ROS elevates the expression of nuclear factor-*κ*B [15], which subsequently translocates to the cell nucleus to activate the expression of inflammatory cytokines, such as tumor necrosis factor-*α* and monocyte chemoattractant protein 1 [16]. These cytokines induce CoNV, recruit inflammatory cells, and further exacerbate inflammation, leading to tissue damage. In this study, we utilized *in vivo* and *in vitro* models to examine whether ROS-mediated angiogenesis plays a vital role in corneal damage. Furthermore, the present study investigated the relationship between G9a and ROS, as well as the potential mechanisms involved in this process.

## 2. Materials and Methods

### 2.1. Antibodies and Reagents

BIX 01294 (BIX) was purchased from Selleck Chemicals Company (Houston, TX, USA). N-acetyl-cysteine (NAC) was supplied by Sigma-Aldrich (St. Louis, MO, USA). The antibodies used in western blotting (WB) experiments, G9a, Nrf2, HO-1, Nox4, vascular endothelial growth factor (VEGF), CD31, CD34, and anti-*β*-actin antibodies were purchased from Abcam.

### 2.2. Experimental Animals and CoNV Model

Male C57BL/6 mice (6–8 weeks old, weight: 20-25 g) were provided by the Experimental Animal Centre of the Medical College of Wuhan University (Wuhan, China). All procedures were performed according to the Guidelines for the Care and Use of Laboratory Animals. All animal experiments were approved by the committee of experimental animals of Wuhan University. All mice were housed at ambient temperature of 20–22°C under a 12-h light/dark cycle, with free access to water and standard chow. CoNV was induced through an alkali burn method as previously reported [17]. All mice were anaesthetized with an intraperitoneal injection of pentobarbital (50 mg/kg). A filtered paper disc (2.5 mm diameter) soaked in sterile sodium hydroxide (1 M) was placed on the central cornea of the right eye for 30 s, and the cornea was immediately rinsed with 0.9% saline solution for 1 min. After the alkali burn, the animals were treated with either dimethyl sulfoxide (DMSO) or different concentrations of G9a inhibitor via subconjunctival injection once daily. The experiment was terminated using euthanasia 1, 4, and 7 days after the alkali burn, and all eyes were enucleated for the following experiment.

All mice were randomly divided into various groups (15 mice per group). In the control group, the right eye was exposed to a filtered paper disc soaked in sterile phosphate-buffered saline. In the alkali burn group, the right eye was exposed to a filtered paper disc soaked in sterile sodium hydroxide and subsequently observed for different periods of time (1, 4, and 7 days). In the alkali burn+BIX group, the mice received BIX (5, 10, and 20 mg/kg) via subconjunctival injection on the day the CoNV model was established. The DMSO group (*n* = 8) was treated with an equal volume of DMSO solution. Following the alkali burn, CoNV was observed using a slit lamp, and neovascularization was quantified and normalized.

### 2.3. Cell Culture and Treatment

Human umbilical vein endothelial cells (HUVECs) were purchased from the American Type Culture Collection (Manassas, VA, USA). HUVECs were cultured in Dulbecco's modified Eagle's medium (DMEM) (Invitrogen) supplemented with 10% fetal bovine serum (FBS), 1% antibiotic solution (penicillin 100 U/ml and streptomycin 100 g/ml) at 37°C, and 5% CO2 in a humidified incubator. The in vitro hypoxia/reoxygenation (H/R) model was established using HUVECs. Briefly, after exposed to hypoxic conditions (1% oxygen and 5% CO2) for 12 h at 37°C in the medium without glucose and serum, the HUVECs were cultured under regular conditions (5% CO2) 24 h with normal medium for reoxygenation.

### 2.4. Real-Time Quantitative Reverse Transcription-Polymerase Chain Reaction (qRT-PCR)

Total RNA from corneal tissues and HUVECs were extracted using RNAiso Plus (Takara Biotech, Dalian, China) according to the manufacturer' s instruction. The purity and concentration of extracted RNA were evaluated by NanoDrop 2000 spectrophotometer (Thermo Scientific, Waltham, MA, USA). 2 *μ*g RNA was reversely transcribed into cDNA using PrimeScript™ RT Reagent Kit (Takara Biotech). The RT-PCR was carried out using IQ SYBR green supermix reagent (Bio-Rad) in accordance to the manufacturer's protocol. The RT-PCR primers designed for specific target genes were as followed. The levels of mRNAs were evaluated according to Ct values and GAPDH served as an internal control.

M-G9a: 5′-CCATCGCTGAGGTCCTTCTGA-3′ (F)

5′-CACGAGACAGGAACAACAGAACA-3′ (R)

M-VEGF-A: 5′-CACTGGACCCTGGCTTTACTG-3′ (F)

5′-CTCAATCGGACGGCAGTAGC-3′ (R)

M-MMP1: 5′-TCCACAGTTGACAGGCTCCG-3′ (F)

5′-GGCACTCCACATCTTGGTTTTC-3′ (R)

M-MMP2: 5′-GACCCTGAAACCGTGGATGAT-3′ (F)

5′-GCCATCAGCGTTCCCATACTT-3′ (R)

M-MMP3: 5′-CTCCACAGACTTGTCCCGTTTC-3′ (F)

5′-GTGCCCTCGTATAGCCCAGAA-3′ (R)

M-MMP9: 5′-AGTTTGGTGTCGCGGAGCA-3′ (F)

5′-AATGGGCATCTCCCTGAACG-3′ (R)

M-CD31: 5′-AAGAAAGGGCTCATTGCGGT-3′ (F)

5′-GCTGTTGGAGTTCAGAAGTGGA-3′ (R)

M-GAPDH: 5′-ATGGGTGTGAACCACGAGA-3′ (F)

5′-CAGGGATGATGTTCTGGGCA-3′ (R)

H-VEGF-A: 5′-GGAGGGCAGAATCATCACGA-3′ (F)

5′-GCTCATCTCTCCTATGTGCTGG-3′ (R)

H-MMP1: 5′-GGACCATGCCATTGAGAAAGC-3′ (F)

5′-TTGTCCCGATGATCTCCCCT-3′ (R)

H-MMP2: 5′-TCAATGGCAAGGAGTACAACAGC-3′ (F)

5′-CACCTTCTGAGTTCCCACCAA-3′ (R)

H-MMP3: 5′-GAGGACACCAGCATGAACCTTG-3′ (F)

5′-CAATCCTGTATGTAAGGTGGGT-3′ (R)

H-MMP9: 5′-TCGACGTGAAGGCGCAGAT-3′ (F)

5′-AGAAGCGGTCCTGGCAGAAATA-3′ (R)

H-CD31: 5′-TTCAACAGAGCCAACCACGC-3′ (F)

5′-CTCCGATGATAACCACTGCAATAA-3′ (R)

H-GAPDH: 5′-TCAAGAAGGTGGTGAAGCAGG-3′ (F)

5′-TCAAAGGTGGAGGAGTGGGT-3′ (R)

### 2.5. WB

The total proteins were extracted from corneal tissues and HUVECs, using the Bicinchoninic Acid Protein Assay Kit (Beyotime) to determine the concentration of protein. The protein was separated in 10% sodium dodecyl sulfate-polyacrylamide gel electrophoresis and transferred to polyvinylidene difluoride membranes (Millipore). Then it was blocked with 5% nonfat dry milk in tris-buffered saline (TBS). Subsequently, membranes were incubated with the primary antibodies at the following dilutions: anti-*β*-actin (1 : 1,000), G9a (1 : 1,000), VEGF (1 : 1000), Nox4 (1 : 1,000), Nrf2 (1 : 1,000), and HO-1 (1 : 1,000) under 4°C overnight. After washed with TBS/Tween 20 twice, the membranes were further immunoblotted with secondary antibody for 1 h at room temperature, followed by washes with TBS/Tween 20 and detection through enhanced chemiluminescence. Protein bands were quantitated through densitometry using the ImageJ software (National Institutes of Health, Bethesda, MD, USA).

### 2.6. Scratch Wound Healing Assay

Briefly, HUVECs (5 × 10^5^ cells) were seeded onto 24-well plates and were cultured until 100% confluence. A sterile pipette tip was used to make a scratch in the monolayer perpendicularly across the centre of the well. The plates were washed with warm PBS twice and then under H/R condition with or without G9a inhibitor, BIX. Images were taken at 0 and 24 h after scratch wounding with an Olympus Microscope. Mitomycin (1.0 mg/ml; Sigma-Aldrich) was applied to exclude cell proliferation influence.

### 2.7. Transwell Migration Array

HUVECs (5 × 10^4^ cells of each chamber) were starved overnight and added to the upper chamber, and the lower chamber was filled with completed medium with or without G9a inhibitor under normal condition or H/R condition. After 24 h, cells were immersed into 3% paraformaldehyde for 15 min, stained with crystal violet, and counted under a light microscope.

### 2.8. Histological Staining

Haematoxylin-eosin staining was performed on sections (4-*μ*m thick) of corneal tissue fixed in 4% paraformaldehyde and embedded in paraffin. Two pathologists who were experienced in ophthalmology and blinded to the group assignments determined the morphological changes.

### 2.9. Immunohistochemistry

The 4-*μ*m paraffin sections were deparaffinized, hydrated, and microwave-repaired. We removed endogenous peroxidase using 3% hydrogen peroxide and added anti-G9a, CD31, and CD34 primary antibody. The reaction was performed in a 37°C reheating box for 2 h and allowed to react overnight at 4°C. Biotinylated secondary antibody and horseradish peroxidase were added and reacted with 3,3′-diaminobenzidine. A positive reaction was observed using a microscope, followed by washing with water to stop the reaction.

### 2.10. ROS Production Detection

Intracellular ROS levels were determined using Reactive Oxygen Species Assay Kit (Nanjing Jiancheng Bioengineering Institute, Nanjing, China) according to the instruction. Briefly, cells pre-treated with different reagents were incubated with 20 *μ*M dichloro-dihydro-fluorescein diacetate (DCFH-DA) in Hanks' balanced salt buffer for 30 min at 37°C. The ROS level was quantified using Flow cytometry (FACSCalibur; BD Biosciences, San Jose, CA, USA).

### 2.11. Tube Formation Assay

HUVECs (1 × 10^3^ cells per well) were cultured and seeded onto the Matrigel (BD Biosciences, CA, USA) and placed under hypoxic conditions for different periods of time. The formation of capillary-like tubes was assessed using an inverted microscope (Olympus, Japan).

### 2.12. Cell Viability

HUVECs (5 × 10^3^ cells) were cultured and seeded onto the 96-well plates. Cell viability was detected using a commercial kit (Beyotime) according to the instructions provided by the manufacturer and evaluated through measurement of absorbance at 450 nm using a microplate reader (Thermo Fisher Scientific).

### 2.13. Luciferase Reporter Assay

The Nox4 promoter reporter vector was designed and synthesized by Sangon Company (Shanghai, China). The Nox4 promoter reporter construction was transiently transfected with control plasmid using Lipofectamine 3000 reagent according to the instructions provided by the manufacturer. Briefly, HUVECs were transfected with plasmid for 6 h; subsequently, the medium was replaced with DMEM/F12 containing 0.2% FBS. After transfection (48 h), HUVECs were exposed to the H/R condition. The luciferase activity was detected using a dual-luciferase reporter system (Promega, Madison, WI, USA).

### 2.14. Adenoviral Infection

Overexpression of Nox4 was established via infection of cells (70–80% confluency) with adenovirus at a multiplicity of infection of 50 in DMEM/F12 without serum, penicillin, or streptomycin for 6 h. Subsequently, the medium was replaced with DMEM/F12 containing 10% FBS for 72 h.

### 2.15. Small Interfering RNA (siRNA) Transfection

HUVECs were transfected with either small interfering RNA against targeting gene or with nontargeting siRNAs (Santa Cruz, CA, USA) at a concentration of 100 nM, and nontargeting siRNAs served as a negative control (NC) for 48 h using Lipofectamine 3000 reagent (we followed the methods of Changhui Diao). The effects of siRNA were assessed using western blot or RT-PCR.

### 2.16. Measurement of MDA and SOD

The SOD and MDA detection kits were purchased from the Nanjing Jiancheng Bioengineering Institute (Nanjing, China). They were used to determine the level of MDA and SOD activities in cell lysates. The blank group was used as the control according to the manuscript instructions in SOD and MDA measurements.

### 2.17. Detection for the Production of H_2_O_2_

Briefly, H_2_O_2_ production was measured by Amplex Red in HUVECs cultured in DMEM containing 0.2% FBS. The HUVECs were treated with or without H/R process for 24 h. For specific cases, HUVECs were pretreated with BIX 01294, G9a siRNA, or Nox4 siRNA and then treated with or without H/R process. To detect the corneal tissue H_2_O_2_ levels, the corneas were first perfused with Hepes-modified Tyrode's solution and homogenized. The hydrogen peroxide in the homogenate was measured using Amplex Red (100 *μ*M, Invitrogen) with 10 U/ml horseradish peroxidase. Fluorescent readings were obtained from the rat cornea after 1 h of incubation at 37°C, and the values were normalized to the protein amount as measured by a Bradford assay. The Amplex Red reagent is a colorless substrate that reacts with H_2_O_2_ with a 1 : 1 stoichiometry to produce the highly fluorescent resorufin (excitation/emission maxima = 570/585 nm).

### 2.18. Statistical Analysis

The statistical analysis was performed using the GraphPad Prism version 5.0 software (GraphPad Software Inc., USA). All values were expressed as the mean ± SD. One-way analysis of variance and the Student-Newman-Keuls test were performed to analyze differences among experimental groups. *P* < 0.05 denoted statistical significance.

## 3. Results

### 3.1. G9a Regulated Hypoxia/Reoxygenation-Induced Proliferation, Migration, and Oxidative Stress In Vitro

**Figure 1:**
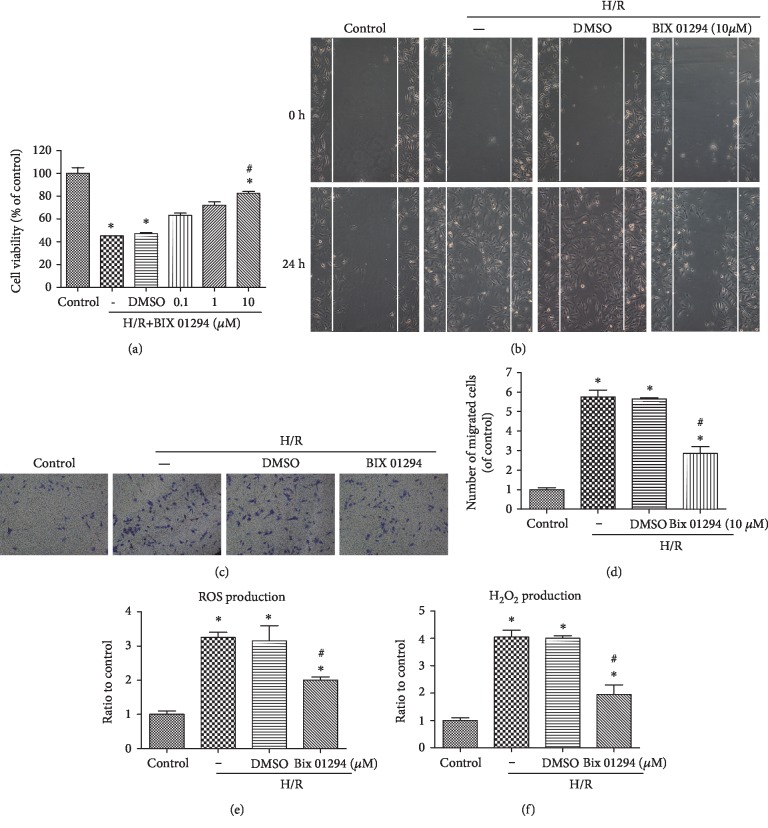
G9a regulated hypoxia/reoxygenation-induced proliferation, migration, and oxidative stress in HUVECs. Cultured HUVECs were treated with different concentrations of BIX 01294 (0.1–10 *μ*M, application 2 h prior to H/R) under normal condition or H/R condition, and cell viability (a) was tested by CCK8 assay. Cell migration was also tested by scratch wound healing (b) and Transwell (c,d) assays. ROS production and H_2_O_2_ production were measured after H/R process with treatment with G9a inhibitor or not. Data are expressed as means ± SD (*n* = 5). ^∗^*P* < 0.05 versus control; ^#^*P* < 0.05 versus H/R. Experiments were repeated 3 times.

Firstly, we determined whether different concentrations of G9a inhibitor affected cell viability subjected to H/R process (Figure 1(a)). The results indicated that cell viability decreased after H/R, but different concentrations of G9a inhibitor could elevate HUVECs cell proliferation, with more obvious effect observed at 10 *μ*M. Scratch wound healing results showed that after H/R, migration of HUVECs was significantly increased (Figure 1(b)). Pretreatment with G9a inhibitor largely attenuated H/R-induced HUVECs migration. Transwell assay results also indicated that H/R largely increased HUVECs migration, which was again inhibited by G9a inhibitor (Figures 1(c) and 1(d)). Furthermore, H/R process-induced elevation of ROS production (Figure 1(e)) and H_2_O_2_ production (Figure 1(f)) in HUVECs was markedly inhibited by G9a inhibitor. Therefore, G9a inhibitor, BIX 01294, displays positive relation in suppressing H/R-induced HUVECs proliferation, migration, and oxidative stress.

### 3.2. Angiogenesis Induced by H/R Depends on Oxidative Stress in HUVECs

**Figure 2:**
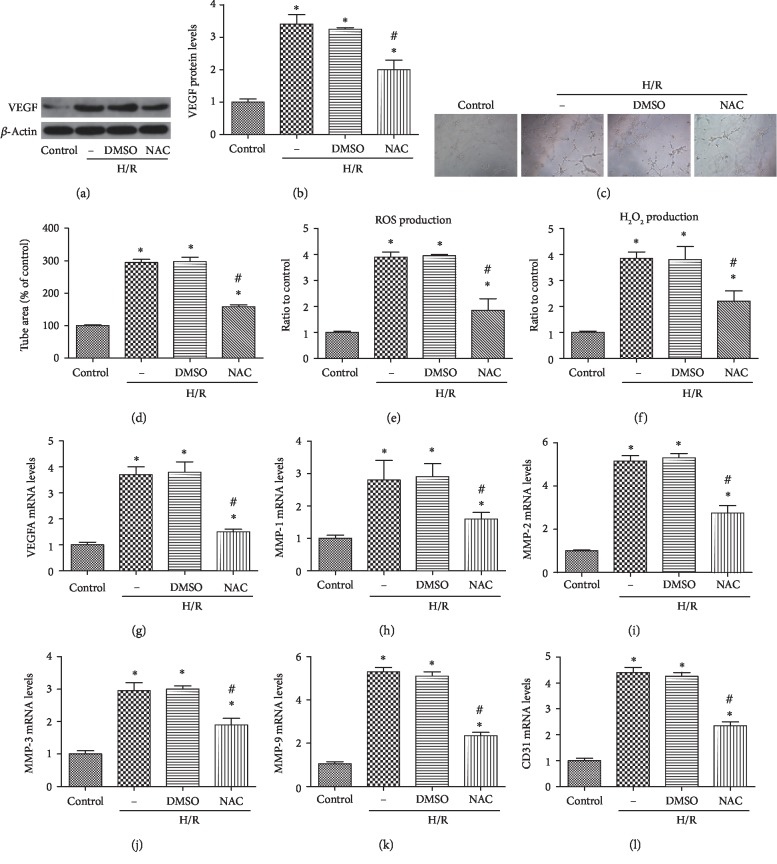
Angiogenesis depended on oxidative stress in HUVECs under the H/R model. Cultured HUVECs were treated with NAC or DMSO under H/R condition, and NAC(1 mM) or DMSO was added 10 min prior to H/R. The expression of VEGF (a,b) was detected by western blot. Serum-starved HUVECs were seeded onto the Matrigel (c,d) and incubated after H/R with or without NAC treatment. ROS production (e) and H_2_O_2_ production (f) were measured after treatment with NAC under H/R. The mRNA levels of proangiogenic factors were also determined by quantitative real-time PCR (g-l). Data are expressed as means ± SD (*n* = 5). ^∗^*P* < 0.05 versus control; ^#^*P* < 0.05 versus H/R. Experiments were repeated 3 times.

Firstly, WB results indicated that VEGF expression in the H/R group was significantly elevated compared with that observed in the control group, but NAC, a ROS scavenger, could reduce the increased VEGF expression induced by H/R (Figures 2(a) and 2(b)). We also performed a tube formation assay, and the results showed that the number of capillary-like tubes was elevated in the H/R group compared with the control group; however, NAC could inhibit the increased tube formation ability induced by H/R (Figures 2(c) and 2(d)). In addition, we found that total ROS production and H_2_O_2_ production were significantly increased in HUVECs upon H/R, which were reversed by treatment with NAC (Figures 2(e) and 2(f)). Besides, NAC reduced the elevated mRNA levels of proangiogenic factors during H/R, including VEGF-A, MMPs (MMP-1, MMP-2, MMP-3, and MMP-9), and cluster of differentiation 31 (CD31) (Figures 2(g) and 2(l)).

### 3.3. Inhibition of G9a Decreased Nox4-Mediated Angiogenesis and Oxidative Stress in HUVECs

**Figure 3:**
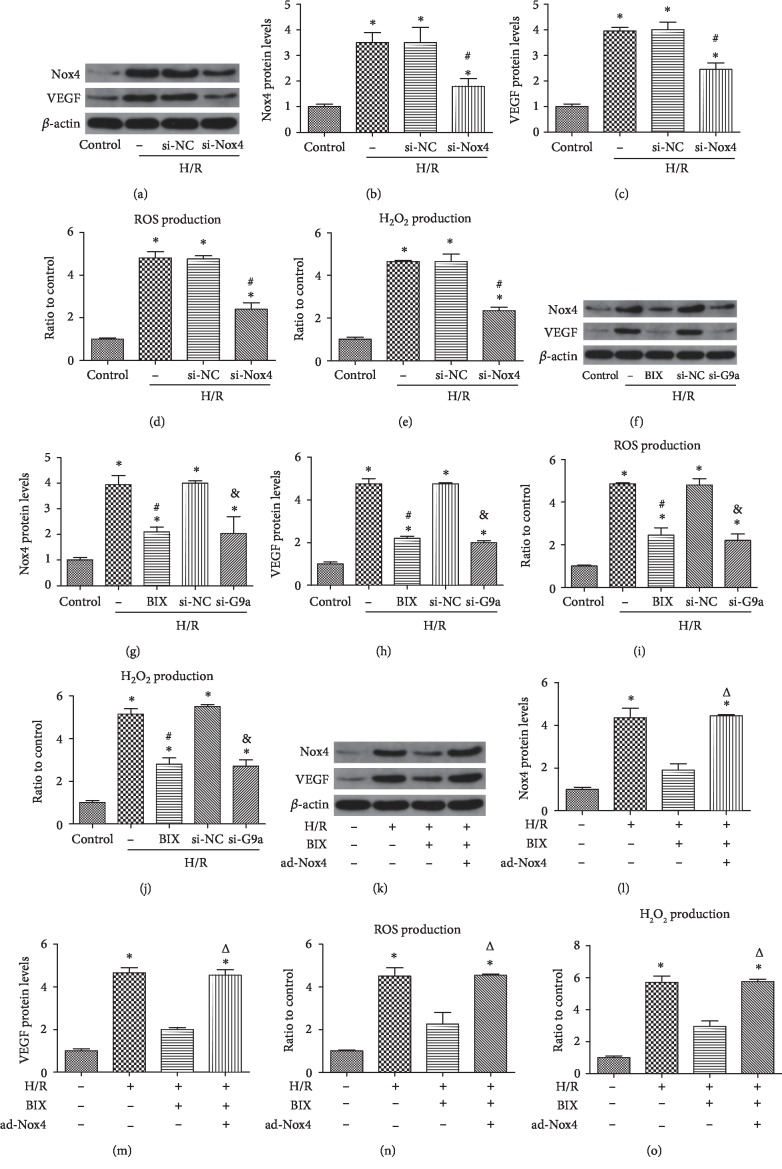
Inhibition of G9a alleviated angiogenesis and oxidative stress through reduction of Nox4. Western blotting analysis for the expression of Nox4 and VEGF after transfection with an si-Nox4 (a-c). ROS production was measured after transfection with an si-Nox4 (d). H_2_O_2_ production was measured after transfection with an si-Nox4 (e). The expression of Nox4 and VEGF was evaluated through western blot after treatment with BIX (10 *μ*M, application 2 h prior to H/R) or transfection with siRNA against G9a (f-h). ROS production was measured after treatment with BIX or transfection with siRNA against G9a (i). H_2_O_2_ production was measured after transfection with an si-G9a (j). HUVECs were treated with 10 *μ*M BIX for 2 h and then infected with an adenovirus carrying the human Nox4 for 48 h, prior to exposure to the H/R process. Western blotting analysis for the expression of Nox4 and VEGF in different groups (k-m). (l,m) The production of ROS (n) and H_2_O_2_ (o) was measured in different groups. Data are expressed as means ± SD (*n* = 5). ^∗^*P* < 0.05 versus control; ^#^*P* < 0.05 versus H/R; ^&^*P* < 0.05 versus H/R+si-NC. Experiments were repeated 3 times.

NADPH oxidases (Nox) family is the prominent source of ROS generation, mainly including Nox1, Nox2, and Nox4. We found that the Nox1 and Nox2 expression levels were unaffected by H/R injury in HUVECs, whereas Nox4 expression was obviously increased under H/R. Then, WB analysis showed reductions in the levels of Nox4 (Figures 3(a) and 3(b)) and VEGF (Figures 3(a) and 3(c)) expression after knockdown of Nox4 under H/R, as well as the production of ROS (Figure 3(d)) and H_2_O_2_ (Figure 3(e)). These findings demonstrated the vital role of Nox4 in H/R-induced angiogenesis and oxidative stress. Subsequently, we detected the effects of G9a on the regulation of Nox4 expression. As shown, treatment with BIX and knockdown of G9a inhibited the H/R-induced expression of Nox4 (Figures 3(f) and 3(g)) and VEGF (Figures 3(f) and 3(h)), as well as ROS production (Figure 3(i)) and H_2_O_2_ production (Figure 3(j)). Furthermore, we compensated the BIX-mediated reduction in Nox4 by delivering an adenovirus carrying human Nox4 to further test whether G9a regulated angiogenesis and oxidative stress through Nox4. The compensation of Nox4 reversed the BIX-mediated reduction of Nox4 (Figures 3(k) and 3(l)) and VEGF expression (Figures 3(k) and 3(m)), as well as the production of ROS (Figure 3(n)) and H_2_O_2_ (Figure 3(o)) in HUVECs under H/R. Therefore, it shows that inhibition of G9a plays antioxidative stress and antiangiogenesis roles through downregulation of Nox4.

### 3.4. G9a Regulated the Expression of Nox4 through the Nrf2/HO-1 Pathway

**Figure 4:**
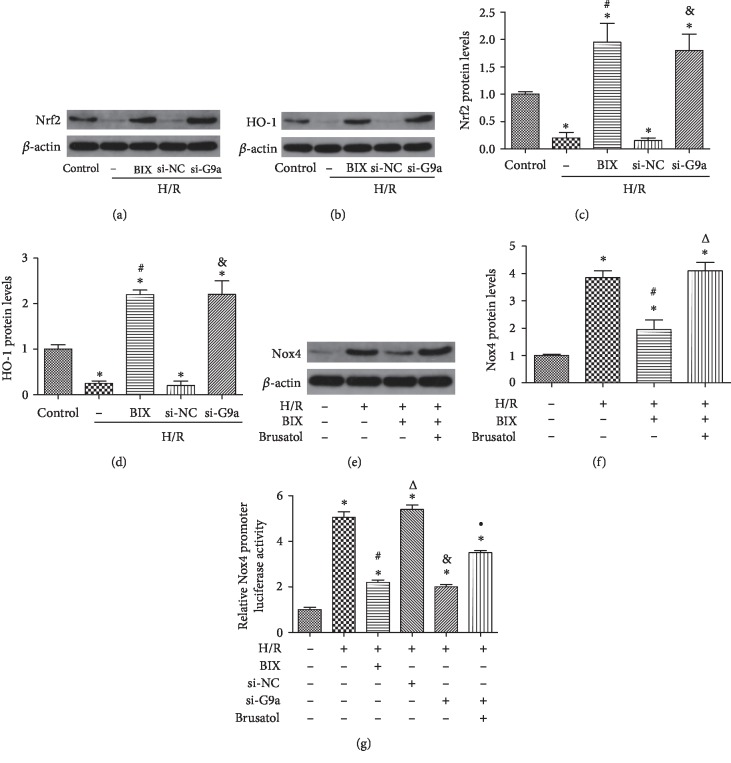
G9a regulated the expression of Nox4 via the Nrf2/HO-1 pathway. Western blotting analysis for the expression of Nrf2 (a) and HO-1 (b) after treatment with BIX (10 *μ*M, application 2 h prior to H/R) or si-G9a and quantification of their expression in fold change (c,d) after treatment with BIX or si-G9a. Combination of BIX (10 *μ*M) and Brusatol (40 *μ*M), application 2 h prior to H/R, reversed the decreased expression of Nox4 (e,f) in HUVECs upon H/R. Luciferase assay to determine the activity of the Nox4 promoter with BIX or si-G9a or combined with Brusatol. Data are expressed as means ± SD (*n* = 5). ^∗^*P* < 0.05 versus control; ^#^*P* < 0.05 versus H/R; ^&^*P* < 0.05 versus H/R+si-NC; ^△^*P* < 0.05 versus H/R+BIX; ^•^*P* < 0.05 versus H/R+si-G9a. Experiments were repeated 3 times.

We also investigated the possible mechanism involved in the regulation of Nox4 by G9a. The Nrf2/HO-1 pathway has been shown to regulate the expression of Nox4. The ratio of Nrf2 (Figures 4(a) and 4(c)) and HO-1 (Figures 4(b) and 4(d)) was decreased after H/R; this effect was partially reversed by treatment with BIX or si-G9a. Brusatol, the inhibitor of Nrf2, was used to further demonstrate the relationship between Nox4 and Nrf2/HO-1. The results showed that the combination of BIX and Brusatol could reverse the reduction of Nox4 expression in HUVECs subjected to H/R (Figures 4(e) and 4(f)). Next, we examined whether inhibition of G9a alleviated Nox4 promoter activity through luciferase reporter detection. HUVECs were transfected with a plasmid containing the promoter region of the human Nox4 gene. The results indicated that either BIX or si-G9a blocked the activity of the Nox4 promoter (Figure 4(g)). However, the combination of si-G9a and Brusatol could reverse the decreased Nox4 activity, demonstrating that G9a regulated Nox4 partially through the Nrf2/HO-1 pathway and transcriptionally decreased the activity of the Nox4 promoter.

### 3.5. Expression of G9a Was Upregulated after Alkali Burn on the Cornea

**Figure 5:**
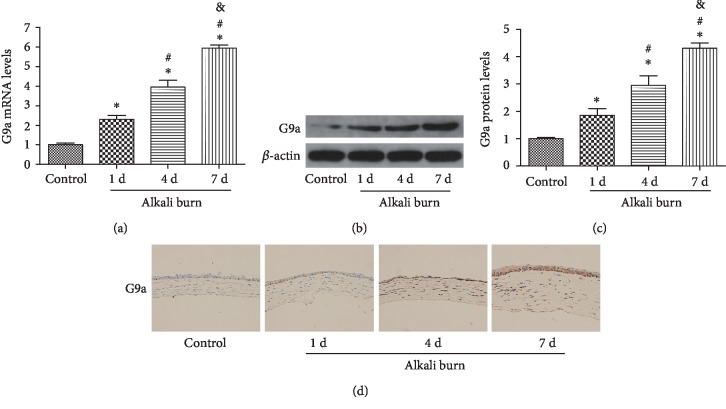
G9a was elevated in alkali burn-injured cornea. The levels of G9a were evaluated through RT-PCR (a) analysis at different postburn days. The levels of G9a protein (b) were assessed at different postburn days, and quantification of G9a expression (c) was determined in fold change relative to the control group. Immunohistochemical staining (d) of G9a in corneal tissues at 1, 4, and 7 days after the alkali burn. Data are expressed as means ± SD (*n* = 5). ^∗^*P* < 0.05 versus control; ^#^*P* < 0.05 versus alkali burn 1d; ^&^*P* < 0.05 versus alkali burn 4d. Experiments were repeated 3 times.

The expression of G9a was initially determined 1, 4, and 7 days after the alkali burn using PCR (Figure 5(a)) and WB (Figures 5(b) and 5(c)). With the extension of the postburn time, the expression of G9a was markedly increased, particularly after alkali burn 7 days. In addition, immunohistochemical staining indicated that G9a was mainly located in the nucleus, and its expression was elevated at day 7 after the alkali burn (Figure 5(d)). These results suggested that G9a might be involved in the development of alkali burn-induced injury. The 7 days postburn time point was selected for the following experiments.

### 3.6. Inhibition of G9a Attenuated Corneal Injury Induced by Alkali Burn

**Figure 6:**
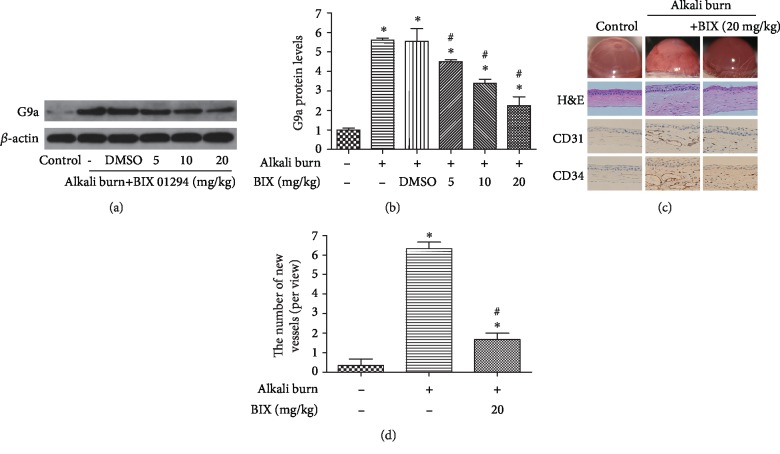
Treatment with G9a inhibitor for corneal injury induced by the alkali burn 7d. Expression of G9a (a) after treatment with BIX 01294 in mice at concentrations of 5, 10, and 20 mg/kg once daily and quantification of G9a expression in fold change (b) relative to the control group. The cornea picture using a slit lamp, H&E staining, IHC staining for CD31, and CD34 (c), and the number of new vessels (d) were detected after the alkali burn 7 days. Data are expressed as means ± SD (*n* = 5). ^∗^*P* < 0.05 versus control; ^#^*P* < 0.05 versus alkali burn. Experiments were repeated 3 times.

BIX was used for the inhibition of G9a. WB indicated that BIX could reduce the expression of G9a (Figures 6(a) and 6(b)), with the most pronounced inhibitory effect observed at the concentration of 20 mg/kg. In parallel with this effect, morphological results of the cornea using the slit lamp and H&E staining showed that the alkali-burned cornea had extensive pathologic vessel growth (Figures 6(c) and 6(d)); however, inhibition of G9a via subconjunctival injection obviously alleviated alkali burn-induced CoNV, which was demonstrated by the reduction in neonatal corneal vessels. Also, the immunohistochemical staining showed that G9a inhibition treatment could reverse the increased CD31 and CD34 positive expression induced by alkali burn. These results demonstrated that inhibition of G9a markedly attenuated corneal injury induced by alkali burn.

### 3.7. Inhibition of G9a Reduced Angiogenesis and Oxidative Stress Induced by Alkali Burn in the Cornea

**Figure 7:**
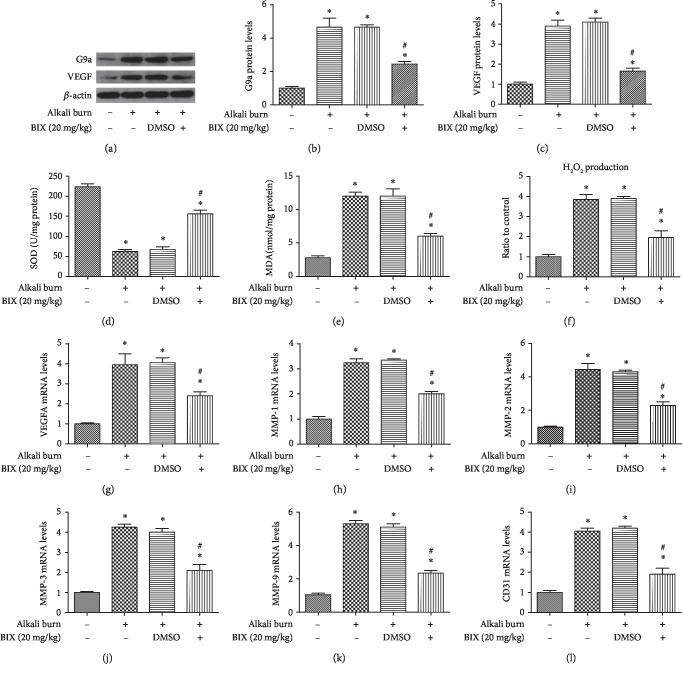
Treatment with BIX 01294 reduced angiogenesis and oxidative stress induced by alkali burn in the cornea. Western blot was performed for the expression of G9a and VEGF (a-c) after treatment with BIX (20 mg/kg, once daily) and quantification of their expression in fold change relative to the control group. SOD activity (d), MDA content (e), and H2O2 production (f) were also detected after treatment with BIX. The mRNA levels of proangiogenic factors were also determined by quantitative real-time PCR (g-l). Data are expressed as means ± SD (*n* = 5). ^∗^*P* < 0.05 versus control; ^#^*P* < 0.05 versus alkali burn. Experiments were repeated 3 times.

The WB results indicated that treatment with a G9a inhibitor via subconjunctival injection could reduce G9a (Figures 7(a) and 7(b)) and VEGF expression (Figures 7(a) and 7(c)) induced by the alkali burn in the cornea. Furthermore, the decreased SOD level (Figure 7(d)), the increased MDA level (Figure 7(e)), and H_2_O_2_ production (Figure 7(f)) induced by alkali burn could be reversed by inhibition of G9a. The mRNA levels of pro-angiogenic factors, including VEGF-A, MMP-1, MMP-2, MMP-3 and MMP-9, and CD31, were elevated after alkali burn 7d (Figures 7(g)–7(i)); however, inhibition of G9a could alleviate mRNA levels of these proangiogenic factors. These results suggested that inhibition of G9a alleviated neovascularization and oxidative stress in the alkali-burned mouse cornea.

### 3.8. Inhibition of G9a Attenuated the Expression of Nox4 and the Nrf2/HO-1 Pathway In Vivo

**Figure 8:**
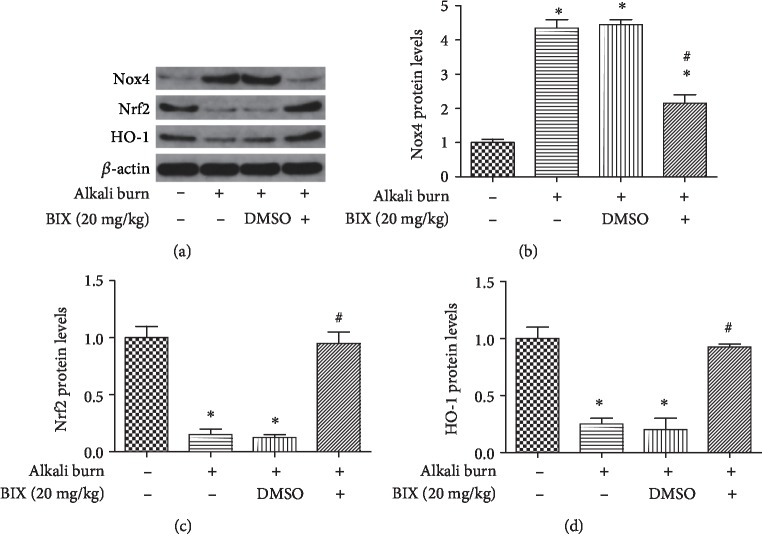
BIX 01294 attenuated Nox4 through Nrf2/HO-1 pathway in mice. Western blotting analysis for the expression of Nox4 and Nrf2/HO-1 after treatment with BIX (20 mg/kg, once daily) and quantification of their expression in fold change relative to the control group (a-d). Data are expressed as means ± SD (*n* = 5). ^∗^*P* < 0.05 versus control; ^#^*P* < 0.05 versus alkali burn. Experiments were repeated 3 times.

The effects of G9a inhibition on expression of Nox4 and the Nrf2/HO-1 pathway observed in vitro also needed to be verified in vivo. As shown in Figure 8, the increased expression of Nox4 (Figures 8(a) and 8(b) was inhibited by treatment with BIX via subconjunctival injection. Meanwhile, the decreased expression of Nrf2 (Figures 8(a) and 8(c)) and HO-1 (Figures 8(a) and 8(d)) in alkali burn model could be reversed by BIX. In summary, these results indicated that inhibition of G9a may reduce the expression of Nox4 through activation of the Nrf2/HO-1 pathway.

## 4. Discussion

In the present study, we focused on the role of G9a in a classical CoNV model and explored the underlying mechanisms. We determined the role of G9a in the corneal alkali burn model and found that inhibition of G9a alleviated alkali burn injury-mediated growth of neonatal corneal vessels in mice. Of note, angiogenesis induced by H/R stimulation relied on oxidative stress in HUVECs, and inhibition of G9a using either a chemical inhibitor or siRNA blocked the Nox4-mediated generation of ROS. Furthermore, the protein levels and transcriptional activity of Nox4 were modulated by G9a through the Nrf2/HO-1 pathway. Therefore, our findings demonstrate that G9a may be a therapeutic target for CoNV, while BIX 01294 may be an effective therapeutic agent for corneal injury.

Among ophthalmic diseases, CoNV is one of the major causes of vision loss, even blindness. Alkali burns cause severe and irreversible damage to the human cornea, provoking the overproduction of numerous angiogenic and inflammatory responses. These factors induce multiple cellular signaling cascades and eventually lead to CoNV [18, 19]. Consistent with these findings, our research demonstrated that 7 days after the alkali burn, corneal oedema and CoNV could be easily observed in the mouse cornea.

It has been reported that G9a, a histone methyltransferase, regulates numerous biological process, included apoptosis, proliferation, migration, and neovascularization. Chen et al. reported that G9a induced numerous angiogenic factors that included angiogenin and interleukin-8 in cancer cells and suppression of G9a could both reduce angiogenic factor expression [20]. Oh et al. found that inhibition of G9a could reduce HIF-1*α* stability and VEGF-induced angiogenesis through the VEGFR-2 signaling pathway in hepatocellular carcinoma cells [21]. Ueda et al. [22] also found that G9a interacted with Jmjd1a to drive mutually opposing expression of the antiangiogenic factor genes accompanied by changes in H3K9 methylation status in cancer cells. The regulation of G9a upon angiogenesis mainly focus on cancer cells; however, the role of G9a in corneal neovascularization still remains unknown. In this study, we firstly observed that alkali burn-induced injury could elevate the expression of G9a. In addition, treatment with the G9a inhibitor BIX 01294 could alleviate the growth of neonatal corneal vessels and infiltration of inflammatory cells, as well as reduce the expression of VEGF, CD31, and CD34. Consistent with our in vivo findings, the in vitro results also indicate that inhibition of G9a alleviated the H/R-induced the expression of proangiogenic factors.

H/R is a main cause of excessive oxidative stress and leads to accumulation of ROS, which creates irreversible cellular damages [23]. Previous study has been found that ROS was the prime initiators of the angiogenic response after alkali injury of the cornea, and its production was immediately enhanced after alkali burn injury in the cornea [24]. Zhang et al. [25] reported that hypoxia induced miR-21 and inhibited SPRY2, thereby enhancing proangiogenic signaling and promoting wound healing in corneal epithelial cells. Other papers [26–28] also found that inhibition of hypoxia inducible gene could decrease corneal neovascularization. The findings of our study are consistent with those previously reported, showing that the expression of angiogenetic factors was increased in H/R-induced HUVECs, as demonstrated by the upregulation of VEGF expression and formation of capillary-like tubes. Meanwhile, the elevated level of angiogenesis could be reversed by treatment with NAC, an ROS scavenger, indicating that H/R-induced angiogenesis depends on oxidative stress in vitro. Furthermore, treatment with the G9a inhibitor BIX 01294 attenuated corneal damage induced by the alkali burn. This effect was attributed to the declined levels of oxidative stress and neovascularization. Consistent with in vivo data, our in vitro research revealed that inhibition of G9a using BIX 01294 or siRNA could alleviate H/R-induced oxidative stress and angiogenesis in HUVECs.

NADPH oxidases (Noxs) family is recognized as the important source of ROS generation, which induces cellular oxidative stress and neovascularization. Previous study reported that Nox4 plays a role in alkali burn-induced corneal neovascularization through regulation of oxidative stress. The inhibition of Nox4 effectively attenuated alkali burn-induced ROS production and mRNA level of proangiogenic factors in the corneas. [29]. Our study was consistent with previous findings, demonstrating the vital role of Nox4 in the regulation of angiogenesis and ROS production induced by H/R. Treatment with BIX 01294 and knockdown of G9a inhibited the H/R-induced Nox4 expression. Furthermore, as we compensated the BIX 01294-mediated reduction in Nox4 by delivering adenovirus carrying the human Nox4 to HUVECs, the results demonstrated that G9a regulated ROS production and angiogenesis through Nox4. Therefore, inhibition of G9a played antioxidative stress and antiangiogenesis roles through the downregulation of Nox4.

Nrf2/HO-1 pathway is considered as a cytoprotective factor regulating the expression of genes coding for antioxidant, anti-inflammatory, and neovascularization. Previous study provided evidence that activation of the Nrf2/HO-1 pathway could subsequently alleviate oxidative stress-induced angiogenesis in HUVECs [30]. Our study was consistent with previous finding and found that activation of Nrf2/HO-1 pathway through inhibition of G9a could alleviate alkali burn-induced neovascularization. Also our in vitro study demonstrated that inhibiting G9a with BIX or si-G9a elevated the expression of Nrf2/HO-1 pathway, and thus reduced Nox4 expression. Moreover, after treatment of both BIX and Nrf2 inhibitor, Brusatol, it could be identified the vital role of the Nrf2/HO-1 pathway between G9a and Nox4, and G9a transcriptionally decreased the activity of the Nox4 promoter.

## 5. Conclusion

In conclusion, we revealed that inhibition of G9a protects the cornea against alkali burn-induced injury and prevents neovascularization by modulating Nox4-dependent production of ROS via the Nrf2/HO-1 pathway. Overall, these results indicate that G9a may be a novel therapeutic target for CoNV.

## Data Availability

The datasets used and analyzed during the current study are available from the corresponding author on reasonable request.

## Abbreviations

G9a: euchromatic histone lysine methyltransferase 2
H3K9: histone H3 lysine 9
CoNV: corneal neovascularization
HUVECs: human umbilical vein endothelial cells
BIX 01294: BIX
siRNA: small interfering RNA
H/R: hypoxia/reoxygenation
ROS: reactive oxygen species
Nox4: NADPH oxidase 4
Nrf2: nuclear factor erythroid-2-related factor 2
HO-1: heme oxygenase-1
H_2_O_2_: hydrogen peroxide
NAC: N-acetyl-cysteine
SOD: superoxide dismutase
MDA: malondialdehyde
DMSO: dimethyl sulfoxide;
DMEM: Dulbecco's modified Eagle's medium
FBS: foetal bovine serum
CO2: carbon dioxide
WB: western blot
qRT-PCR: real-time quantitative reverse transcription-polymerase chain reaction
H&E staining: haematoxylin-eosin staining.
